# A hunter virus that targets both infected cells and HIV free virions: Implications for therapy

**DOI:** 10.1186/1742-4682-9-52

**Published:** 2012-12-06

**Authors:** Cody Greer, Gisela García-Ramos

**Affiliations:** 1 Department of Biology, 101 Morgan Bldg, University of Kentucky, Lexington, KY, 40506, USA; 2 Current address: Division of Biology and Biomedical Sciences, Washington University, St. Louis, MO, 63110, USA

**Keywords:** Engineered therapeutic virus, Antiviral virus, Virus-virus fusion, Viral dynamics, HIV super-infection, Mathematical model

## Abstract

The design of ‘hunter’ viruses aimed at destroying human immunodeficiency virus (HIV) infected cells is an active area of research that has produced promising results *in vitro*. Hunters are designed to target exposed viral envelope proteins in the membranes of infected cells, but there is evidence that the hunter may also target envelope proteins of free HIV, inducing virus-virus fusion. In order to predict the effects of this fusion on therapy outcomes and determine whether fusion ability is advantageous for hunter virus design, we have constructed a model to account for the possibility of hunter-HIV fusion. The study was based on a target cell-limited model of HIV infection and it examined the hunter therapeutic effect on recovering the HIV main target cells, the activated CD4^+^ T lymphocytes. These cells assist in setting up an immune response to opportunistic infections. The study analyzed the hunter dual mechanisms to control infection and because of diverse estimates for viral production and clearance of HIV, simulations were examined at rates spanning an order of magnitude. Results indicate that without hunter-HIV fusion ability, hunters that kill HIV-infected cells lead to a substantial recovery of healthy cell population at both low and high HIV turnover rates. When hunter-HIV fusion is included, cell recovery was particularly enhanced at lower HIV turnover rates. This study shows that the fusion ability, in addition to hunter infection ability, could be a favorable attribute for improving the efficacy of hunter-viral therapy. These results provide support for the potential use of engineered viruses to control HIV and other viral infections.

## Introduction

Currently, HIV-1 therapy involves long-term treatment with highly active antiretroviral drugs (HAART). Such treatment is effective at reducing viral loads and allowing immune system recovery, but a low level of HIV remains. This viremia may arise from latently infected reservoirs such as resting CD4^+^ T-cells or sanctuary sites where drug penetration is suboptimal [1-3]. These reservoirs have impeded the eradication of HIV [4]. A less toxic alternative may be to control HIV through the use of another virus. Some natural viruses have the ability to slow AIDS disease progress through mechanisms which are not yet clear [5]. For example, infection with GB Virus C following HIV infection often slows HIV disease progress [6]. A step further would be to design a ‘hunter’ virus that specifically targets HIV infection. Ideally, this designed virus would coexist long-term with HIV in the body, making multiple treatments unnecessary. Models have been proposed for the use of an engineered defective interfering virus (DIV) as well as for a hunter virus to treat HIV infection [7-10]. Such models may provide an important insight into the dynamics of a hypothetical infection and elucidate the attributes of an optimal therapeutic virus.

This study builds on previous models of hunter virus super-infection [8,10]. A hunter virus is designed to infect and kill only HIV-infected cells. Its engineered envelope contains HIV-1 host cell receptors such as CD4 and CXCR5. This allows the hunter to bind to the HIV-1 Env proteins which are present in the membrane of HIV-1 infected cells [11]. After binding, the hunter virus infects the cell and rapidly reproduces causing the cell lysis. A critical feature of the therapeutic virus is that it can replicate and sustain the therapeutic infection. Models have shown that a hunter could be very effective if designed with high infection and production rates relative to HIV-1 [8]. Experimental precedents exist for the creation of such a virus from a vesicular stomatitis virus (VSV) recombinant [11,12]. Hunter virus design is still an active area of research; a highly efficient hunter virus was recently created using a similar approach to Schnell et al. [11] but additionally incorporating human OX40 ligand in the hunter membrane [13]. These engineered viruses were very effective *in vitro*, but none have yet been tested *in vivo*.

The model presented in this study takes some elements from previous models [8,10] and adds an additional ability of the hunter virus to fuse with free HIV virions. Virus-virus fusion has been shown to occur between modified HIV particles or VSV and HIV [14,15], and the resulting fused viral particles were infectious to the hosts of both component particles. Hunter-HIV fusion was also suggested with a hunter derived from VSV [11] because of the occurrence of cells that were positive for VSV proteins but contained undetectable levels of HIV proteins. Schnell et al. proposed that since the hunter cannot enter a cell without the help of HIV-1 Env proteins, the two viruses may have entered together as a fused virus, allowing the hunter to stop HIV production before it started. Thus, it is likely that a hunter virus attaches to HIV-1 Env proteins on either infected cells or free HIV virions. In the case of hunter attachment to free HIV virions, the resulting fused particles have membrane receptors capable of infecting either of the cell targets of the component virions: susceptible healthy CD4^+^ cells or HIV-infected cells.

The model examined in the present study accounts for this possibility which has not been considered in previous hunter models. A therapeutic virus with two targets could significantly change the dynamics of the system and result in either increased or decreased healthy cell recovery, making virus-virus fusion either a goal or an obstacle in future therapeutic virus design. The purpose of this study is to use computer simulation of an infection system to evaluate activated healthy CD4^+^ cell recovery (and thus therapeutic potential) in a hunter virus therapy targeted to both HIV-infected cells and free HIV virions. The fusion between different types of viruses may have practical applications, such as in the development of a HIV vaccine [14].

### Model

#### Model description

**Figure 1:**
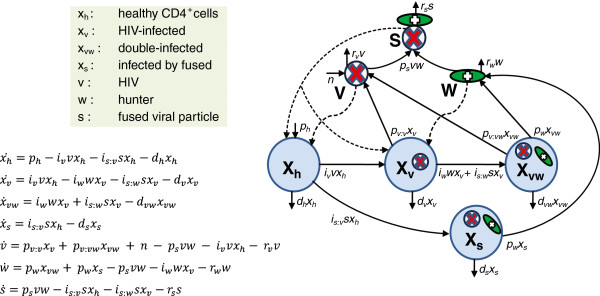
**Schematic diagram and model formulation.** The elements of the model consist of cells susceptible to viral infections (activated CD4^+^ cells, in large circles), HIV virions (small circles) and hunter viruses (ovals). The diagram illustrates the dynamics of susceptible cells in healthy condition *x*_*h*_ and the formation of infected cell populations (HIV-infected *x*_*v*_ and double infected *x*_*vw*_, *x*_*s*_) via pathogen or therapeutic viral infection (HIV *v*, hunter *w*, and HIV-hunter fused particles *s*). Dashed arrows indicate the cell population targeted by each type of virus. In the diagram, healthy cells *x*_*h*_ infected by HIV are transferred to the population of HIV-infected cells *x*_*v*_, and then release HIV virions. Alternatively, healthy cells *x*_*h*_ can be infected by fused particles *s* to become *s*-infected cells *x*_*s*_ that release hunters. Hunters *w* and fused particles *s* can infect HIV-infected cells and generate double-infected cells *x*_*vw*_, which release both HIV and hunter virions. The graph also shows the formation of fused particles *s*. Parameters describe infection rates *i*, death rates of cells *d*, removal rates of viruses *r*, and production rates *p*. Subscripts denote to which population a particular parameter applies (see parameters in Table 1). Equations (denoted as Eqs. 1 in Model description) describe the change in the number of cells or virions over time *t* (e.g. *x*^·^_*h*_ = *dx*_*h*_/*dt*). The positive and negative terms at the right side of a given equation correspond to incoming and outcoming arrows, respectively, for the relevant population in the diagram.

This study uses a target cell limited model for representing HIV infection [16], and it is based on García-Ramos et al. (2003) [8]. HIV-1 preferentially replicates in activated CD4^+^ T cells [1,2]. A typical healthy individual has about 1000 CD4^+^ cells/μL in peripheral blood, but only a fraction are activated CD4^+^ cells and thus susceptible of HIV infection. The present model represents HIV infection and therapeutic infection using differential equations (Eqs. 1). The equations describe the changing population densities of healthy target cells *x*_*h*_ which are activated CD4^+^ cells, three types of infected cells, and three viruses consisting of HIV-1 *v*, hunters *w* and HIV-hunter fused particles *s* (see diagram in Figure 1). In this infection system, populations of cells and viruses are impacted by various processes of birth and death. Thus, each term in a given equation represents a process which adds or subtracts cells or virions in proportion to the rate constant for the process and the densities of the populations participating in the process. Rates are written in terms of production rates *p*, death rates of cells *d*, infection rates *i*, and removal rates of viruses *r*. Subscripts denote to which population a particular parameter applies (e.g. *p*_*h*_ refers to the production rate of healthy cells; *p*_*v:v*_ and *p*_*v:vw*_ denote HIV production by *x*_*v*_ and *x*_*vw*_ cells, respectively). See Table 1 for a full description of each parameter, its default value, and references. With these conventions in mind, we can follow the events described by the equations.

**Table 1:** Parameter values and initial variable values

	**Parameter**	**Description**	**Value**	
			**Default HIV turnover**	**High HIV turnover**
Healthy cell parameters	*p*_*h*_	Production rate of healthy cell ^1^ (cd^−1^)	2	2
	*d*_*h*_	Death rate of healthy cell ^2^ (d^−1^)	0.01	0.01
HIV related parameters	*i*_*v*_	Infection rate of HIV ^3^ (v^-1^d^-1^)	0.004	0.004
	*p*_*v:v*_	HIV production rate in an HIV-infected cell ^4^ (vc^-1^d^-1^)	50	50×10
	*p*_*v:vw*_	HIV production rate in a double-infected cell ^†^ (vc^-1^d^-1^)	5	5×10
	*r*_*v*_	Removal rate of HIV virions ^5^ (d^−1^)	3	3×10
	*d*_*v*_	Death rate of HIV-infected cell ^6^ (d^−1^)	0.33	0.33
	*n*	Constant influx of HIV ^7^ (vd^−1^)	0.84	0.84×10
Hunter related parameters	*i*_*w*_	Infection rate of hunter ^8^ (v^-1^d^-1^)	0.02	0.02
	*p*_*w*_	Production rate of hunter ^9^ (vc^-1^d^-1^)	1800	1800
	*r*_*w*_	Removal rate of hunter virions ^8^ (d^−1^)	2	2
	*d*_*vw*_	Death rate of a double-infected cell ^8^ (d^−1^)	2	2
Fused particle related parameters	*p*_*s*_	Formation rate of fused viruses ^†^ (v^-1^d^-1^)	0.02	0.02
	*i*_*s:v*_	Infection rate of healthy cells by *s*^†^ (v^-1^d^-1^)	0.5×*i*_*v*_	0.5×*i*_*v*_
	*i*_*s:w*_	Infection rate of HIV-infected cells by *s*^†^ (v^-1^d^-1^)	0.5×*i*_*w*_	0.5×*i*_*w*_
	*r*_*s*_	Removal rate of fused viruses ^†^ (d^−1^)	3	3×10
	*d*_*s*_	Death rate of a cell first infected with *s*^†^ (d^−1^)	*d*_*vw*_	*d*_*vw*_
	Variable		Initial values	
Interacting populations	*x*_*h*_	Healthy CD4^+^ cells (c)	10	
	*x*_*v*_	HIV infected cells (c)	1	
	*x*_*vw*_	Double infected cells (c)	0	
	*x*_*s*_	Cells infected by *s* (c)	0	
	*v*	HIV virions (v)	0.001	
	*w*	Hunter virions (v)	0.001	
	*s*	Fused virions (v)	0	

Units: d, day; number of cells or virions per μL (c, v, respectively). References: ^1^[8,17]; ^2^[18,19]; ^3^[20-22]; ^4^[1,23-25]; ^5^[23,26-28]; ^6^[18,26,29]; ^7^[30]; ^8^[10]; ^9^[11]; ^†^Inferred (see Model section).

The model assumes that healthy target cells *x*_*h*_ are produced at a constant rate *p*_*h*_ (Eqs. 1; Figure 1). These cells are targets of infection by the population of HIV virions *v*. This infection by HIV at rate *i*_*v*_ reduces the population of healthy cells and creates infected cells *x*_*v*_ at a rate proportional to the populations of HIV and healthy cells. HIV is then produced by infected cells at a rate *p*_*v:v*_, proportional to the infected cell population. The therapeutic hunter virus *w* behaves similarly except that its target is the HIV-infected cell population *x*_*v*_. Infection by hunter at rate *i*_*w*_ creates double-infected cells *x*_*vw*_ at a rate proportional to the densities of hunter viruses and HIV-infected cells. Double-infected cells then produce hunter virus at a rate *p*_*w*_ and HIV at a rate *p*_*v:w*_. Thus the hunter is capable of hijacking an HIV-infected cell to produce more hunter viruses. Populations of cells are also diminished by inherent rates of cell death *d*_*h*_, *d*_*v*_ and *d*_*vw*_.

The model presented here includes several key differences from the 2003 model [8]. The most important is the ability of free HIV and hunter to fuse at rate *p*_s_, creating a new population of fused virions *s* that can infect both healthy CD4^+^ cells and HIV-infected cells at rates *i*_*s:v*_ and *i*_*s:w*_, respectively. The healthy cells infected by fused viral particles are denoted by *x*_*s*_, and HIV-infected cells infected by fused particles become double-infected cells similarly to those infected by the hunter alone (*x*_*vw*_). The equations are described below,

(1) $$\begin{array}{c} {\dot{x}}_{h}=p_{h}-i_{v}vx_{h}-i_{s:v}sx_{h}-d_{h}x_{h}\\ {\dot{x}}_{v}=i_{v}vx_{h}-i_{w}wx_{v}-i_{s:w}sx_{v}-d_{v}x_{v}\\ {\dot{x}}_{\mathrm{vw}}=i_{w}wx_{v}+i_{s:w}sx_{v}-d_{\mathrm{vw}}x_{\mathrm{vw}}\\ {\dot{x}}_{s}=i_{s:v}sx_{h}-d_{s}x_{s}\\ \dot{v}=p_{v:v}x_{v}+p_{v:\mathrm{vw}}x_{\mathrm{vw}}+n-p_{s}\mathrm{vw}-i_{v}vx_{h}-r_{v}v\\ \dot{w}=p_{w}x_{\mathrm{vw}}+p_{w}x_{s}-p_{s}\mathrm{vw}-i_{w}wx_{v}-r_{w}w\\ \dot{s}=p_{s}\mathrm{vw}-i_{s:v}sx_{h}-i_{s:w}sx_{v}-r_{s}s \end{array}$$

The present model includes other differences from the 2003 model [8] which are summarized below: (1) The addition of a constant influx *n* of free HIV virions from other sources in the body to represent a reservoir of HIV [10]. (2) The addition of a small amount of HIV production *p*_*v:vw*_ from cells infected by hunters [10]. (3) Cells infected by fused virus particles *x*_*s*_ do not produce HIV [11], and cell death occurs at rate *d*_*s*_. (4) When a virus infects a cell, the model now accounts for the loss of that virus from the free viral population. This applies to HIV, hunter, and fused viruses, therefore, the populations of viruses are reduced via cell infection and by removal of free virions *r*_*v*_, *r*_*w*_, *r*_*s*_. (5) Modifications of default parameter values based on more recent estimates [10].

Thus this study differs from the 2003 model by considering the hunter’s fusion ability as well as refining the more fundamental equations and updating parameters values. To isolate the effect of fusion we compare the full model with a fusion-less version of itself. Fusion can easily be removed from the model by setting the rate of production of fused particles *p*_*s*_ to zero. The system of differential equations was analyzed numerically using the ode45 solver from MATLAB (version 7.14; MathWorks, Inc.).

#### Model evaluation

Most of the model’s parameters were either found experimentally or inferred from previous studies (see references in Table 1). However, estimates of five new default parameter values for the fused particle were required, and assumptions for inferring these values are described below.

(1) *p*_*s*_: formation rate of fused virus particles. There is no literature describing rates of virus-virus fusion. This process requires binding of hunter to HIV-1 Env proteins, which also occurs when the hunter infects cells. Therefore the rate was initially assumed equal to the rate of infection by the hunter virus. Since infection and fusion are otherwise different processes, a wide range of production rates was tested (± 50% default value).

(2) *r*_*s*_: removal rate of fused virus particles. Assumed equal to HIV-1 removal. This may be an overestimate since fusion may leave less surface area of HIV exposed for removal mechanisms than in a free particle. Hunter has a lower removal rate than HIV since it carries an envelope with human proteins.

(3) *d*_*s*_: death rate of a previously healthy cell infected by *s*. Assumed equal to death rate of double-infected cells by default. However, the life cycle of these cells may differ because HIV may have not altered the cell's structure or function. Perhaps this causes a change in the speed that a hunter can replicate and kill the cell. Therefore, a range of values was tested.

(4–5) *i*_*s:v*_ and *i*_*s:w*_: infection rate of healthy cells and HIV-infected cells by fused particles, respectively (see illustration of hunter-HIV fusion in Additional file 1). We assumed that this fused particle has a fraction of receptors for each viral component available for cell infection. Therefore, default was assumed to be lower than the infection rate by single particles.

**Figure 2:**
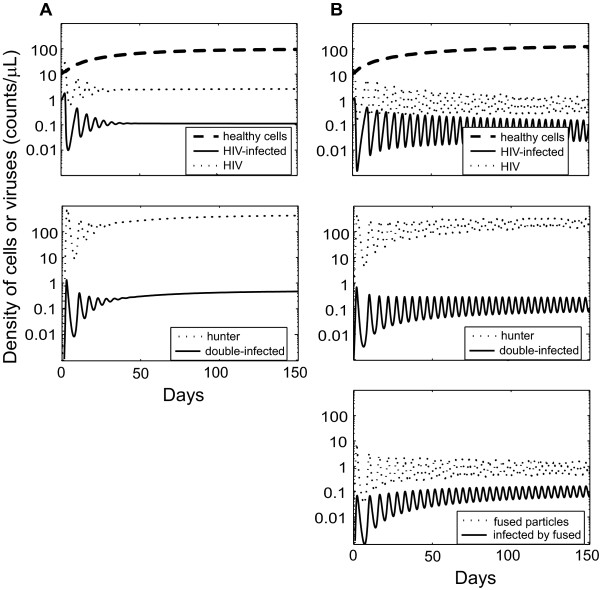
**Infection dynamics for both types of hunters.** (**A**) Hunter virus only targets HIV-infected cells (updated 2003 model). (**B**) Hunter virus double targeted (fusion model). First 150 days after hunter introductions. Healthy CD4^+^ cell count in both models continues to increase until reaching equilibrium by day 400 in the updated 2003 model and a limit cycle of negligible amplitude by day 700 in the fusion model. In the fusion model, the other populations also show cyclic behaviors. For exact densities, see Table 2. Values of the parameters are in Table 1 .

**Figure 3:**
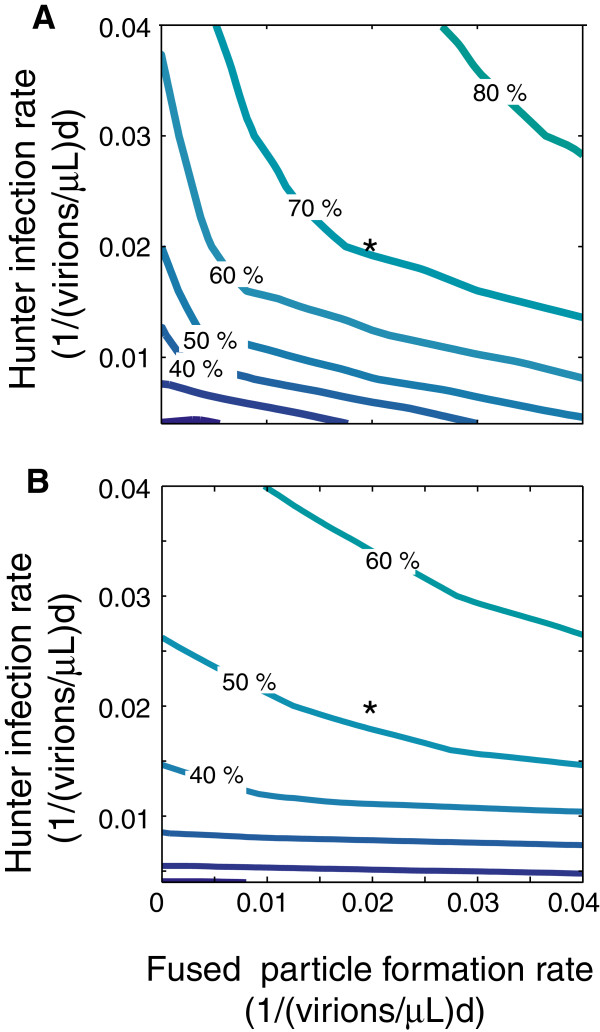
**Influence of hunter infection rate and fused particle fusion rate on therapeutic efficacy.** Each line represents the combinations of hunter infection and fused particle formation rates which yield a particular percent of cell recovery. (**A**) At default HIV turnover rate. Raising hunter infection rate alone increases recovery faster than raising fused particle production rate alone, but optimal recovery is achieved by increasing both parameters. Recovery increases steeply at lower values and then begins to level off. (**B**) At high HIV turnover rate (10×default). Fusion rate has very little influence on recovery at low hunter infection rates, but the therapeutic benefit of hunter-HIV fusion improves greatly at high hunter infection rates. The asterisk (*) represents cell recovery at the default combination of parameter values (Table 1).

**Figure 4:**
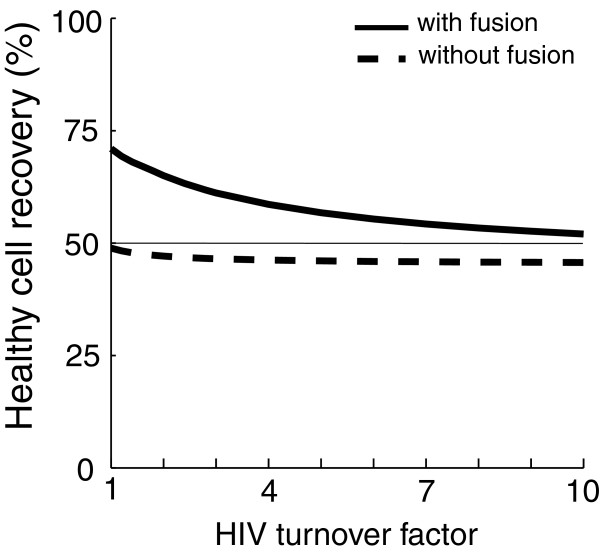
**Effect of HIV turnover rate on therapy efficiency.** Recovery decreases as HIV turnover rate increases. The decrease is steeper for a fusion-capable hunter, decreasing (but not eliminating) its advantage over fusion-incapable hunter at high turnover rates. Within this range, the fusion model displays periodic oscillations at lower HIV turnover rates (<1.7× default). Otherwise, the models reach a stable equilibrium.

To evaluate the model, we first determined the percentage of recovery of healthy cells for the default parameter values (Figure 2, Table 2). Because parameter estimates for the therapeutic infection rely on inference rather than experimental data, we performed sensitivity analyses that estimated cell recovery when each of the parameters in the model was subjected to a ± 50% change in value (Table 3). We then examined the effect of simultaneously manipulating both cell infection rates by fused particles *s* (Table 4); as well as the concerted effects of fused particle formation and hunter infection rates on cell recovery (Figure 3). In addition, these analyses were also assessed for the overall effect of HIV turnover rates on the hunter therapeutic efficacy (Table 2; Figure 4).

**Table 2:** Equilibrium densities for the populations of cells and virions

	**Default parameters (Low HIV turnover)**		**High HIV turnover**	
	**No fusion**	**Fusion**^**a**^	**No fusion**	**Fusion**
Healthy cells (% Recovery)	98 (49%)	141.8-142.2 (71%)	91 (45.5%)	104 (52%)
HIV-infected cells	0.11	0.03-0.12	0.11	0.1
HIV	2.6	0.31-1.0	3	2
Hunter	443	180-340	472	408
Double-infected cells	0.49	0.09-0.25	0.52	0.41
Fused virus particles *s*	–	0.52-1.4	–	0.55
Infected by fused *s*	–	0.08-0.17	–	0.06

^a^Interval values for stable oscillations obtained at about 700 days. Units: counts of cells or viruses per μL.

**Table 3:** Parameter sensitivities

**Parameter**	**Parameter change from default ± 50%**	**% Recovery of healthy cells at low HIV turnover**	**% Recovery of healthy cells at high HIV turnover**
Default	0	71	52
*i*_*v*_	+	61	42
	–	80	69
*p*_*v:v*_	+	62	45
	–	78	63
*p*_*v:vw*_	+	71	49
	–	70	55
*r*_*v*_	+	74	61
	–	67	40
*d*_*v*_	+	72	52
	–	71	52
*n*	+	71	51
	–	68	53
*i*_*w*_	+	76 (+5)	58 (+6)
	–	55	37
*p*_*w*_	+	79 (+8)	61 (+9)
	–	47	35
*r*_*w*_	+	59	43
	–	83 (+12)	66 (+14)
*d*_*vw*_	+	64	44
	–	77 (+6)	57 (+5)
*p*_*s*_	+	74 (+3)	54 (+2)
	–	66	49
*i*_*s:v*_	+	71	52
	–	71	52
*i*_*s:w*_	+	71	52
	–	71	52
*r*_*s*_	+	70	52
	–	71	52
*d*_*s*_	+	66	51
	–	79 (+8)	55 (+3)

The percent of healthy CD4^+^ cell recovery caused by changing model parameters values at low (default) and high (10×default) HIV turnover values. Results were similar for different initial values of the variables. Note: The parameters *p*_*h*_ and *d*_*h*_ were not tested for sensitivity because they define the base healthy CD4^+^ cell concentration of 200 cells/μL to which all other results are compared. Parenthesis indicates the additional recovery in relation to default for hunter related parameters.

**Table 4:** **Sensitivity of CD4**^**+**^**cell recovery to infection rates of fused particles *s***

**Infection rates (μL/virion day)**	***i***_***s:v***_**= *i***_***v***_	***i***_***s:v***_**= 0.5**×***i***_***v***_	***i***_***s:v***_**= 0**	***i***_***s:v***_**= 0**	***i***_***s:v***_**= 0.5×*i***_***v***_
	***i***_***s:w***_**= *i***_***w***_	***i***_***s:w***_**= 0.5×*i***_***w***_ *	***i***_***s:w***_**= 0**	***i***_***s:w***_**= 0.5×*i***_***w***_	***i***_***s:w***_**= 0**
Healthy cells at default HIV turnover (cells/μL)	142 (71%)	142 (71%)	139 (70%)	142 (71%)	139 (70%)
Healthy cells at high HIV turnover (cells/μL)	104 (52%)	104 (52%)	104 (52%)	104 (52%)	104 (52%)

*: default.

Note: Percentage indicates the recovery relative to the maximum healthy level.

## Results

Without HIV infection, the model predicts a maximum density of susceptible healthy cells that we referenced as 100% healthy cell level. This maximum density was 200 cells/μL, and this particular value agrees with previous infection models based on activated CD4^+^ cells [16,20]. Because activated cells are a fraction of the CD4^+^ cell population, we discuss the results in terms of percent healthy level rather than cell density. With HIV infection only, healthy cell density at equilibrium is reduced to 2.5% of the healthy level (${\hat{x}}_{h}=5\;\text{cells}/\text{μL}$). In order to assess the effect of hunter fusion, results were obtained for a hunter that targeted only HIV-infected cells as well as for a hunter that targeted both HIV-infected cells and free HIV virions. Because different techniques have yielded a variety of estimates for viral production and clearance of HIV [23,26,27], the same simulations were run at lower and higher rates of HIV turnover. A summary of these results and a full list of population densities at equilibrium for each scenario are included in Table 2.

When the hunter virus targeted HIV-infected cells only, the healthy cell population recovers to 49% of the normal level (${\hat{x}}_{h}=98\;\text{cells}/\text{μL}$). When the hunter also fused to free HIV virions, the healthy cell population recovered to 71% of the normal level (${\hat{x}}_{h}=142\;\text{cells}/\text{μL}$). Thus, the targeting of free HIV by the hunter significantly improves the effectiveness of therapy at default parameter values. The two scenarios showed distinctive behaviors consisting of equilibrium (without fusion) and limit cycles (with fusion), which were reached at different speeds but the general dynamic is clearly seen within the first 150 days (Figures 2A, B). In a limit cycle, a variable continuously changes value through time but returns to the same value at fixed time intervals. In the fusion model, the healthy cell population exhibited a limit cycle with negligible amplitude of oscillation, while the other populations continued to oscillate at larger amplitudes. Despite these oscillations the free HIV concentration stayed well below that of the model without fusion. Simulations were run for several initial conditions besides those in Table 1, and the system reached the same equilibrium (without fusion) or limit cycles (with fusion; Figures 2A, B), suggesting that the dynamics were globally stable. The appearance of oscillatory dynamics reflects the larger HIV control by a default hunter with fusion ability. Notice that the dynamical behavior in the fusion model may change with values of the parameters. For example at high HIV turnover rate values (Table 1) the fusion model showed equilibrium instead of limit cycles (Table 2).

Estimates of HIV production and removal rates can be greater than the default values [24,27,28]. Therefore, the simulation was run at default rates as well as at a range of HIV production and removal rates up to ten times greater than default (Figures 3A, B and 4). This required raising HIV production *p*_*v:v*_ in infected cells, production *p*_*v:vw*_ in double-infected cells, influx rate *n*, HIV virus removal *r*_*v*_, and fused virus removal *r*_*s*_ (Table 1). The effectiveness of the hunter virus decreased as HIV turnover increased, but the hunter had a significant therapeutic effect even at the highest turnover rates (Figure 4). The decrease in recovery with HIV turnover was much steeper in the fusion model. At ten times default HIV production and removal rates, cells recovered to 46% (${\hat{x}}_{h}=91\;\text{cells}/\text{μL}$) without fusion vs. cells recovered to 52% (${\hat{x}}_{h}=104\;\text{cells}/\text{μL}$) of normal levels with fusion. Thus at larger rate of HIV turnover, fusion ability confers a smaller advantage to the hunter.

### Model sensitivity

An analytical solution to the equilibrium equations could not be found, but a virtual experiment provided insight into the driving force behind the improved recovery of healthy cells in the fusion model. The therapeutic infection was run for different parameter values (± 50% each default value) and results show that recovery was insensitive to fused particle infection rates *i*_*s:v*_ and *i*_*s:w*_, and to removal rate *r*_*s*_ (Table 3). In our previous study [8], the infection rate of the hunter *i*_*w*_ was a critical factor for successful hunter virus treatment, and it is important in this study as well. Variation in HIV infection rate *i*_*v*_ and hunter removal rate *r*_*w*_ also significantly altered the resulting recovery. The production rate *p*_*s*_ of fused particles and the death rate *d*_*s*_ of cells infected by fused virus particles are the most sensitive of the new fusion-related parameters introduced in this study.

### Sensitivity due to fused particle structure: alternative infection rates

A fused particle contains the envelope proteins of both HIV and hunter, so it could potentially infect either healthy cells or HIV-infected cells. The mechanism of fusion and the structure of the resulting particle are unknown, but the model at default parameters assumes that the fused particle envelope maintains spatially separate regions for the envelope proteins of its composite viruses. A simple representation of a fused particle is shown in Figure S1 and it drawn to scale using established dimensions of the spherical HIV virion and cylindrical VSV virion [31,32]. Briefly, the model at default parameter values assumes that each particle has half of its receptors available for binding and infecting its target cell while the other half are blocked by the other virion. Thus the infection rate *i*_*s:v*_ of the fused particle in healthy cells is half of the HIV infection rate *i*_*v*_. Likewise the infection rate *i*_*s:w*_ of HIV-infected cells is half of the hunter infection rate *i*_*w*_. It is also possible that the viruses merge and change conformation, which could lead to uneven ratios of available receptors and thus infection rates. Therefore, the model was also tested in the extreme scenarios. Surprisingly, recovery was unaffected even when the infective ability of fused virus particles was completely removed from the model. This was observed both at default parameter values and with rapid HIV turnover rates (Table 4).

### Formation of multiple fused particles: an alternative model

Models were also created to include the formation of other combinations of fused virus particles (see Additional file 1). Since each HIV virion has several protein spikes capable of binding to CD4 and coreceptors [32], it is possible that more than one hunter might bind to the same HIV particle. Likewise, each hunter has several CD4 molecules in its membrane, so it could bind to multiple HIV [11]. Accounting for these possibilities did not significantly affect cell recovery.

### Accounting for defective HIV particles

Since only a fraction of HIV particles produced is infectious, we modified the model to account for the defective HIV particles that may fuse with the hunter and restrict it from reaching its target (see Additional file 1). We assumed that 10% of HIV particles released is infectious [25]. Surprisingly, healthy cell recovery was unaffected in this scenario. The hunter population remains large enough to effectively control the HIV load.

## Discussion and conclusions

How exactly does the ability of the hunter virus fusion with free HIV result in improved healthy cell recovery? Hunter-HIV fusion may facilitate recovery through both the formation of the fused particle, as well as the ability of the fused particle to infect healthy and HIV-infected cells. Our results indicated that the fused particle’s infection rates proved to have little effect on cell recovery (Table 4). This suggests that the infection of healthy cells by fused particles, which offer an additional and large source of hunters, may be canceled because of the cost of killing these healthy cells. Even though fused particles can kill HIV-infected cells and further reduce HIV load, these outcomes insignificantly affected the recovery of healthy cells under the range of the infection rates tested (Table 4). Enhanced recovery then must result from the fusion process itself, which reduces free HIV load. When HIV fuses with hunter, HIV is no longer able to complete its production/life cycle. Even though HIV can still infect healthy cells, these cells are not releasing HIV virions. Though hunter-HIV fusion caused some loss of hunters, this did not appear to significantly affect hunter density since it was much larger than HIV (Table 2).

Results of this study indicate that hunter virus therapy could be very effective against HIV, especially when the hunter is capable of fusing with free HIV particles. When the hunter was capable of fusion, the model yielded healthy CD4^+^ cell recovery to 71% of normal levels, compared to a 49% recovery in treatment without fusion ability (Table 2). Thus the ability of a hunter to fuse with both target cells and free virus significantly enhances (by 45%) therapeutic outcome when the default parameters were used. The extent of recovery varied under other sets of parameters, with a minimum of 52% recovery seen in simulations with HIV production and removal rates raised by an order of magnitude (Table 2). In all cases tested, however, recovery was better with fusion ability than without (Figures 3A, B and 4, Table 2). Accounting for other possibilities such as multiple fusion events and fusion with non-infectious free HIV did not greatly affect recovery (see Additional file 1).

Which parameter values deserve the attention of a virus designer? As listed in the ‘Parameter estimations’ section of this study, five new parameters were introduced. Decreasing either the death rate *d*_*s*_ of cells infected by fused particles or the death rate *d*_*vw*_ of double-infected cells improves recovery (i.e. a longer life of these cells would produce more hunters, Table 3). The hunter infection rate *i*_*w*_, is a critical parameter and was examined extensively in this study (Figure 3). Without a high hunter infection rate, a higher proliferation rate of the hunters would be required to achieve a significant recovery of the healthy cell population. The most efficient path to recovery appears to be at ratios in which the infection rate of the hunter is slightly higher than the production rate of fused particles (Figure 3). Therefore of the new parameters related to fusion, the death rate *d*_*s*_ and the formation rate *p*_*s*_ of fused particles are for virus designers the most useful and modifiable parameters. Given the broad range of estimates for HIV production and clearance rates, it is important to determine to what extent cell recovery via hunter–HIV fusion depends on these turnover rates. Previous studies found that HIV turnover rate had little effect on the hunter efficiency of killing HIV-infected cells, while larger estimates of HIV clearance tended to cancel larger HIV production resulting in a similar recovery [8,10]. In the present study, a range of HIV production and removal rates spanning an order of magnitude was examined [23,24,27,28]. The overall therapeutic effect decreased as these rates increased, but in all cases recovery was therapeutically significant, and fusion ability conferred an advantage to the hunter virus (Figure 4). Thus the model suggests that hunter virus treatment may offer significant therapeutic benefit even at very high rates of HIV turnover, and treatment is further enhanced when the hunter virus can also fuse to its target free virion.

Admittedly there are several processes left unaccounted for by this model, the most significant of which are the immune response and cell dynamics [10], and the spatial distribution of viruses and cells within the body [33]. However, the exact mechanism of T-cell depletion is still under scrutiny, as is the rate of viral exchange between body compartments [23,34]. In a previous study we evaluated the original hunter in the presence of a target cell life cycle with resting and activated stages along a CTL (cytotoxic T-lymphocyte) immune response [10]. CTL is considered the main immune response against HIV infection via killing infected cells. Therapy efficacy with these refinements [10] was consistent with findings in the 2003 model based on activated CD4^+^ cells and with no CTL [8]. The present model sought to avoid a complex system in favor of simplicity and extrapolation. It can be extrapolated that fusion ability could further aid recovery in the presence of an immune response by creating a state of lowered free HIV density, which has been correlated with reduced apoptosis of healthy cells and reduced overall immune activation [35,36]. On the other hand, the CTL immune response was shown to compete with the hunter virus for HIV-infected cells, and the less efficient competitor was eliminated [10], indicating that this immune response did not persist with effective hunters. This suggests that the present model could be capable of capturing the expected levels of cell recovery without modeling a complex immune response. Also, it is possible that the hunter may have the added benefit of targeting latently infected CD4^+^ cells and significantly reducing the HIV reservoir; however, we suspect that this additional dynamic would not change qualitatively our results.

When considering the results of this study with the 2003 study, the most influential parameters in hunter virus design include: (1) the infection rate of the hunter virus and (2) the formation rate of fused HIV-hunter particles. These processes are comparable to CTL and antibody immune responses, respectively. In this study which featured a low population of infected cells and HIV virions (typical of a chronic HIV infection), the hunter infection rate had a greater effect than fusion rate, and the best results are obtained by a combination of infection and fusion ability (Figure 3). Thus, an ideal hunter virus should both infect and fuse at the highest possible rates. Further *in vitro* testing of the hunter virus will help to quantify the HIV-hunter fusion rate more precisely and identify the attributes determining fusion rate so that this knowledge can be applied in future hunter virus design. Also, *in vivo* testing of the hunter virus could allow an assessment of this model’s prediction regarding effectiveness thresholds for the hunter virus and therapeutic value in the presence of an immune response.

## Competing interests

The authors declare that they have no competing interests.

## Authors’ contributions

The authors contributed equally to this paper. Both authors read and approved the final manuscript.

## Supplementary Material

Additional file 1 A file (3 pages) that includes supplementary material. Click here for file
