# Supplementary material for: A hunter virus that targets both infected cells and HIV free virions: Implications for therapy
Source: Theor Biol Med Model. 2012 Dec 6;9:52. doi: 10.1186/1742-4682-9-52 (PMC3551785; doi:10.1186/1742-4682-9-52)
Supplement: Additional file 1 — A file (3 pages) that includes supplementary material. [file 1742-4682-9-52-S1.pdf]

A hunter virus that targets both infected cells and HIV free virions: Implications for therapy

Cody Greer[1] and Gisela García-Ramos[2]

Department of Biology, 101 Morgan Bldg., University of Kentucky, Lexington KY 40506 USA
[1]codejon@gmail.com; [2]ggarc0@uky.edu

Additional file 1- Supplementary material

A. Formation of multiple fused viral particles

A model was created accounting for up to three combinations of fused virions (*b:* hunter-HIV-hunter, and *c:* HIV-hunter-HIV; see Figure S1; Eqs. S1-8). New production rates and the infection rates were estimated with the same reasoning as discussed in the Model section: a bound particle was modeled as having half of its total receptors available and therefore half of its infection and fusion rate. The central virus was considered completely blocked and incapable of fusion or infection. By this reasoning, particle *b* can infect only HIV-infected cells at a rate $i_b = (0.5 + 0.5) \times i_w = i_w$. Similarly, particle *c* can infect only healthy cells at a rate $i_c = (0.5 + 0.5) \times i_v = i_v$. As explained in the Model section, conformation changes could result in other combinations of infection rates. However, this would not significantly affect results since simulation with default parameters showed that the new particles *b* and *c* added a negligible cell recovery (Table S1). Further, the population densities of particles *b* and *c* were relatively low (Table S1), so more complex fused particles would be even less common since they are formed from these *b* and *c* populations. Therefore, effects from all other fused particle types would be minimal, and further simulation is assumed not necessary.

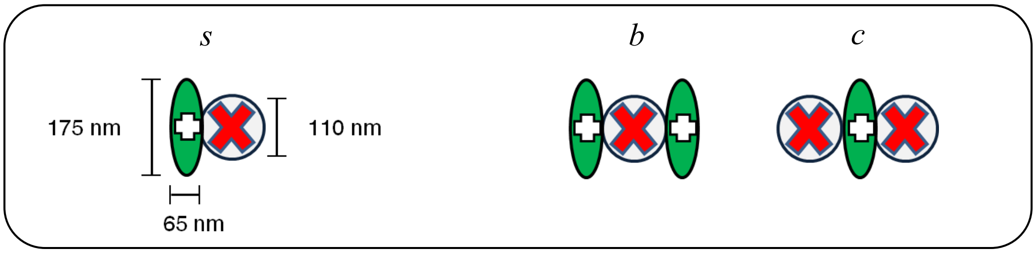

Figure S1 Representation of the combinations of fused viral particles. *s* illustrates the simple fusion of one hunter and one HIV virion (spherical), showing particles sizes [1,2]. *b* and *c*, are combinations of three particles. The illustrations assume that fused viruses may share a membrane without losing individual shapes.

This more complex model (Model S1) has two additional equations respect to the main Model (Eqs. 1) that account for the dynamics of the new fused particles *b* and *c*. Equations were derived using a diagram connecting cells and virions, such as in Figure 1, where incoming and outcoming arrows are positive and negative terms, respectively. There are several differences

relative the main Model, for example, the change in number of healthy and HIV-infected cells (Eqs. S1 and S2) include removals caused by infections of c and b particles, respectively. Notice that $x_{s|c}$ describes the population of cells that are infected with s and c particles.

$$\dot{x_h} = p_h - i_v v x_h - i_{s:v} s x_h - i_c c x_h - d_h x_h \quad \text{(S1)}$$

$$\dot{x_v} = i_v v x_h - i_w w x_v - i_b b x_v - i_{s:w} s x_v - d_v x_v \quad \text{(S2)}$$

$$\dot{x}_{vw} = i_w w x_v + i_b b x_v + i_{s:w} s x_v - d_{vw} x_{vw} \quad \text{(S3)}$$

$$\dot{x}_{s|c} = i_{s:v} s x_h + i_c c x_h - d_{s|c} x_{s|c} \quad \text{(S4)}$$

$$\dot{v} = p_{v:v} x_v + p_{v:vw} x_{vw} + n - p_s v w - p_c s v - i_v v x_h - r_v v \quad \text{(S5)}$$

$$\dot{w} = p_w x_{vw} + p_w x_{s|c} - p_s v w - p_b s w - i_w w x_v - r_w w \quad \text{(S6)}$$

$$\dot{s} = p_s v w - p_c s v - p_b s w - i_{s:v} s x_h - i_{s:w} s x_v - r_s s \quad \text{(S7)}$$

$$\dot{b} = p_b s w - i_b b x_v - r_b b \quad \text{(S8)}$$

$$\dot{c} = p_c s v - i_c c x_h - r_c c \quad \text{(S9)}$$

Table S1 Cell recovery and densities of distinctive fused particles

s (hunter-HIV) (particles/μL)[1]	b (hunter-HIV-hunter) (particles/μL)	c (HIV-hunter-HIV) (particles/μL)	x_h (cells/μL) (% of recovery)
0.4-0.9	0.25-0.73	0.0007-0.004	143 (71.5%)

[1]Model S1 (Eqs. S1-9) was evaluated at default parameter values (Table 1) with new parameters as in Appendix A. It also assumed that removal rates of b and c particles are similar to simple fused particles, $r_b = r_c = r_s$; and production rates are a half of simple fused particles, $p_b = p_c = p_s/2$.

B. Model with presence of noninfectious HIV

About 10% of HIV produced by an infected cell is infectious [3]. Since some of the noninfectious virions produced still have intact Env protein spikes, it was necessary to consider that hunter virus could bind to noninfectious HIV virions, which were not included in the basic model. A model (Model S2) was created that assumed a ratio of noninfectious HIV with intact Env proteins to infectious HIV of 9:1, making the hunter nine times more likely to bind to a noninfectious particle than an infectious one (see Eqs. S10-18). This represented the most extreme scenario in which defective HIV is capable of fusing with the hunter. Model S2 was based on the main model (Eqs. 1) and it has two additional equations for describing the populations of defective-HIV v_D (Eq. S17) and defective-fused particles s_D (Eq. S18). Other differences are the last terms of Eqs. S11, S12 and S15, reflecting the effect of defective particles on the infection system. See footnote in Table S2 for model evaluation.

Thus the overall rate of formation of fused particles was ten times the rate of the basic model and it was expected that the noninfectious HIV particles would act as 'decoys,' diverting the hunter virus from its targets. However, healthy cell recovery was not affected (Table S2). The hunter population was still large enough to control infectious HIV as effectively as in the basic model.

$$\dot{x_h} = p_h - i_v v x_h - i_{s:v} s x_h - d_h x_h \quad \text{(S10)}$$

$$\dot{x_v} = i_v v x_h - i_w w x_v - i_{s:w} s x_v - d_v x_v - i_{s:w} s_D x_v \quad \text{(S11)}$$

$$\dot{x}_{vw} = i_w w x_v + i_{s:w} s x_v - d_{vw} x_{vw} + i_{s:w} s_D x_v \quad \text{(S12)}$$

$$\dot{x}_s = i_{s:v} s x_h - d_s x_s \quad \text{(S13)}$$

$$\dot{v} = p_{v:v} x_v + p_{v:vw} x_{vw} + n - p_s v w - i_v v x_h - r_v v \quad \text{(S14)}$$

$$\dot{w} = p_w x_{vw} + p_w x_s - p_s v w - i_w w x_v - r_w w - f p_s v_D w \quad \text{(S15)}$$

$$\dot{s} = p_s v w - i_{s:v} s x_h - i_{s:w} s x_v - r_s s \quad \text{(S16)}$$

$$\dot{v_D} = 9 \times (p_{v:v} x_v + p_{v:vw} x_{vw} + n) - f p_s v_D w - r_v v_D \quad \text{(S17)}$$

$$\dot{s}_D = f p_s v w - i_{s:w} s_D x_v - r_s s_D \quad \text{(S18)}$$

Table S2: Defective HIV does not significantly affect cell recovery

	Ratio of noninfectious to infectious HIV	Hunter density (particles/μL)[1]	Healthy cell density (cells/μL) (% of recovery)
Basic model	9:1	176-337	141.8-142.1 (71%)

[1]Model S2 was evaluated at default parameter values (Table 1). It assumed that removal rate of defective HIV is equal to normal HIV (r_v); removal rate of defective HIV-fused particles is equal to simple fused particles (r_s); and production rate of defective HIV-fused particles p_{sd} is equal to a fraction f of the simple fused particles ($p_{sd} = 0.9 \times p_s$).

References

1. Howatson AF, Whitmore GF: **The development and structure of vesicular stomatitis virus.** *Virology* 1962, **16**:466–478.

2. Zhu P, Liu J, Bess J Jr., Chertova E, Lifson JD, Grise H, Ofek GA, Taylor K A, Roux KH: **Distribution and three-dimensional structure of AIDS virus envelope spikes.** *Nature* 2006, **441**:847– 852.

3. Dimitrov DS, Willey RL, Sato H, Chang LJ, Blumenthal R, Martin MA: **Quantitation of human-immunodeficiency-virus type-1 infection kinetics.** *J Virol* 1993, **67**:2182–2190.
